# Improving cardiovascular risk stratification through multivariate time-series analysis of cardiopulmonary exercise test data

**DOI:** 10.1016/j.isci.2024.110792

**Published:** 2024-08-22

**Authors:** Evangelos Ntalianis, Nicholas Cauwenberghs, František Sabovčik, Everton Santana, Francois Haddad, Jomme Claes, Matthijs Michielsen, Guido Claessen, Werner Budts, Kaatje Goetschalckx, Véronique Cornelissen, Tatiana Kuznetsova

**Affiliations:** 1 Research Unit Hypertension and Cardiovascular Epidemiology, KU Leuven Department of Cardiovascular Sciences, University of Leuven, Leuven, Belgium; 2 Stanford Cardiovascular Institute and Division of Cardiovascular Medicine, Stanford University School of Medicine, Stanford, CA, USA; 3 Rehabilitation in Internal Disorders, KU Leuven Department of Rehabilitation Sciences, University of Leuven, Leuven, Belgium; 4 Department of Cardiology, Hartcentrum, Virga Jessa Hospital, Hasselt, Belgium; 5 Faculty of Medicine and Life Sciences, Hasselt University, Hasselt, Belgium; 6 Cardiology, KU Leuven Department of Cardiovascular Sciences, University of Leuven, Leuven, Belgium

**Keywords:** Cardiovascular medicine, Kinesiology, Artificial intelligence

## Abstract

Nowadays cardiorespiratory fitness (CRF) is assessed using summary indexes of cardiopulmonary exercise tests (CPETs). Yet, raw time-series CPET recordings may hold additional information with clinical relevance. Therefore, we investigated whether analysis of raw CPET data using dynamic time warping combined with k-medoids could identify distinct CRF phenogroups and improve cardiovascular (CV) risk stratification. CPET recordings from 1,399 participants (mean age, 56.4 years; 37.7% women) were separated into 5 groups with distinct patterns. Cluster 5 was associated with the worst CV profile with higher use of antihypertensive medication and a history of CV disease, while cluster 1 represented the most favorable CV profile. Clusters 4 (hazard ratio: 1.30; *p* = 0.033) and 5 (hazard ratio: 1.36; *p* = 0.0088) had a significantly higher risk of incident adverse events compared to clusters 1 and 2. The model evaluation in the external validation cohort revealed similar patterns. Therefore, an integrative CRF profiling might facilitate CV risk stratification and management.

## Highlights

• Raw CPET data may hold additional information on cardiorespiratory fitness
• Using multivariate time-series analysis, participants were separated into 5 clusters
• Clusters 4 and 5 had a significantly higher risk of incident cardiovascular events
• An integrative cardiorespiratory fitness profiling facilitates risk stratification

## Graphical abstract

**Figure:**
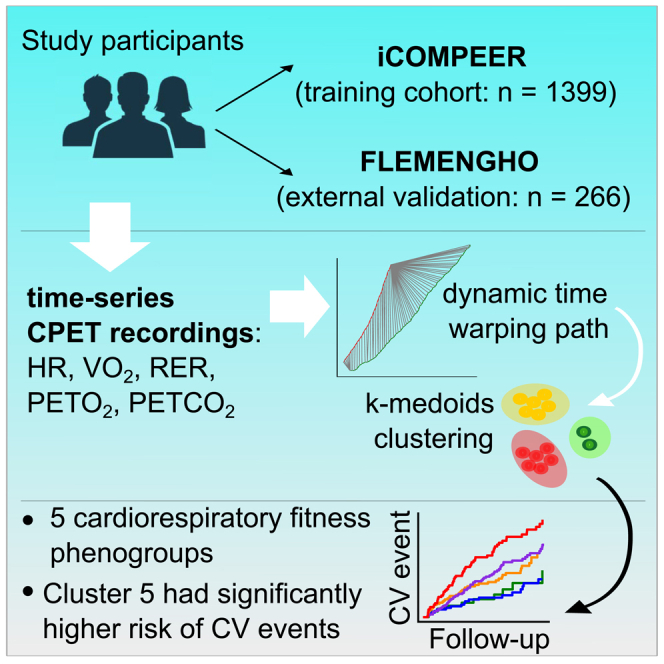

## Introduction

Cardiovascular (CV) disease considerably burdens healthcare systems.^1^^,^^2^ Despite advances in medicine and technology, CV diseases remain the most common cause of mortality and morbidity worldwide.^2^^,^^3^ Therefore, there is still a need for better identification of individuals at high CV risk to efficiently implement risk management strategies. Assessment of cardiorespiratory fitness (CRF) is considered an objective measure of overall health status because it integrates main physiological functions of the respiratory, CV, and musculoskeletal systems.^4^ Previous studies demonstrated that poor CRF is associated with significantly higher risk of developing adverse events.^4^^,^^5^

Cardiopulmonary exercise test (CPET) is the gold standard to assess CRF in clinic, and thus some of the CPET summary metrics may help in CV risk stratification.^4^^,^^6^ On the other hand, CPET tests generate an abundance of temporal data with complex interrelations, which makes their interpretation difficult. The introduction of machine learning (ML) or deep learning algorithms which can extract hidden information from such complex data may aid in exploiting the potency of CPET time series for clinical purposes.^7^^,^^8^ However, ML approaches, both supervised or unsupervised, to evaluate complex cardiorespiratory data to assess CRF and hence CV risk remain to be developed.

Recently, a few studies applied ML approaches to CPET indexes to address different research questions.^9^^,^^10^^,^^11^^,^^12^^,^^13^ For instance, some of these studies utilized supervised ML approaches for predicting hypertension,^9^ diagnosing heart failure^10^^,^^12^ or identifying the exercise limitations.^13^ Two other studies investigated the utility of ML-based interpretation of CPET summary metrics for CV risk stratification in patient cohorts.^14^^,^^15^ However, all these studies investigated the importance of integrative interpretation of summary CPET metrics, while the potentially useful information included in the temporal characteristics of the raw CPET data is yet to be explored.

A few previously published studies investigating time-series CPET data were limited in using supervised learning algorithms for diagnostic purposes^16^^,^^17^ or for predicting the efficacy of exercise programmes.^18^ To our knowledge, no study has yet integrated time-series CPET data into ML-based clinically relevant patient phenogroups for CV risk profiling. Therefore, in this study we investigated whether we could apply an unsupervised ML approach to identify distinct CRF phenogroups from raw CPET recordings.

## Results

### Characteristics of the training cohort

The 1,399 integrated computer modelling of cardiorespiratory fitness for personalized risk profiling (iCOMPEER) participants included 528 (37.7%) women, 839 (60%) patients with history of CV disease, and 998 (71.3%) individuals with hypertension of whom 876 (62.6%) were on antihypertensive medication. Mean age and body mass index of the participants were 56.4 ± 12.9 years and 28 ± 5.1 kg/m^2^, respectively. Table S1 presents the clinical characteristics of participants by sex. Male participants reached significantly higher peak values for almost all summary CPET and spirometry indexes.

### Time-series CPET phenogroups

**Figure 1:**
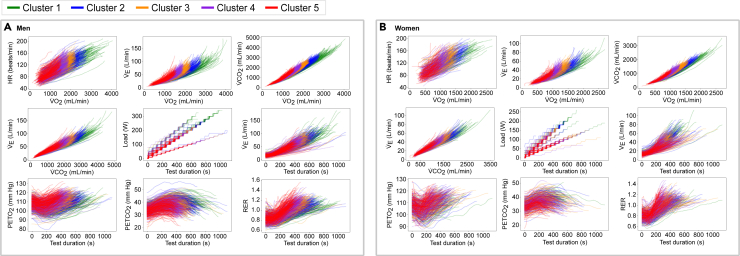
Nine-panel plot showing the clustering results of raw time-series CPET recordings derived by DTW and k-medoids for the training cohort (A and B) (A) shows the results for men, and (B) shows the results for women.

The optimal number of clusters was 5 in men and 4 to 5 in women (Figure S1). For consistency, we opted to perform clustering toward 5 phenogroups in both sexes. Figure 1 shows a modification of the nine-panel plot of the CPET clustering results for men (*panel A*) and women (*panel B*).

**Figure 2:**
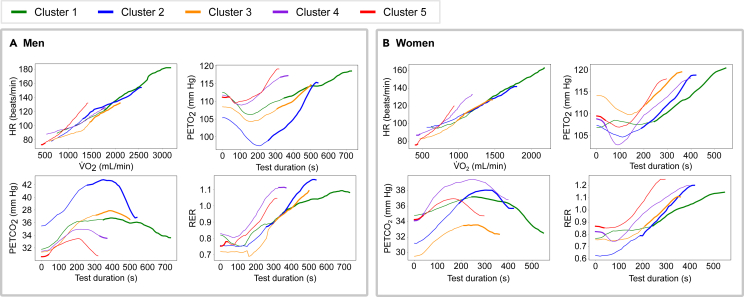
Most important regions of the CPET recordings for cluster assignment (A and B) Interpretation of the clustering results by identifying the most distinctive part of the raw time-series CPET recordings in men (A) and women (B). The individual curves illustrate the most representative CPET recording of each cluster (centroid). The highlighted segments correspond to the most distinctive parts of the curves as derived by the proposed interpretability approach.

Between the clusters we observed significant differences in peak values and in the shape of the CPET curves, especially toward the end of the test (Figure 2). In both sexes, the peak oxygen uptake rate (VO_2_) and heart rate (HR) gradually declined from phenogroup 1 to 5, with phenogroups 4 and 5 exhibiting the lowest values. Regarding end-tidal partial pressure of oxygen (PETO_2_) and end-tidal partial pressure of carbon dioxide (PETCO_2_), phenogroups 1 and 3 presented considerably less steep curves than the other phenogroups, while PETO_2_ and PETCO_2_ values during the test differed between the phenogroups. For respiratory exchange ratio (RER), we observed: (1) a steeper increase in RER during the second half of CPET in phenogroup 3 compared to phenogroup 1 and (2) higher RER throughout the whole CPET examination in phenogroups 4 and 5, reaching their peak RER in a shorter period. The differences in shape of the PETO_2_, PETCO_2_, and RER curves between phenogroups suggest that not only peak values but also curve shape contributed to the clustering.

### Interpretability of clustering on CPET time series

The most distinctive part of the curves for assigning individuals to phenogroups 1, 2, and 3 lay at the end of the test for both men (Figure 2A) and women (Figure 2B). This could be attributed to both the differences in the values for VO_2_ and HR and the differences in the shape of the curves of PETO_2_, PETCO_2_, and RER as described earlier. For assignment to phenogroup 4, the most distinctive phase was the later phase of the test in men and the beginning of the test in women. In phenogroup 5, in both men and women, the part of the PETO_2_, PETCO_2_, and RER curves at the beginning of the test was the most distinguishable from the curves assigned to the other phenogroups. This might be attributed to the fact that the participants assigned to the cluster 5 had a considerably lower VO_2_ and HR (and the women also higher RER) at the beginning of the test.

### Clinical validation of CPET phenogroups

#### Association of CPET phenogroups with CV risk factors

Table 1 shows the clinical and summary CPET characteristics of the participants by phenogroup. The participants’ CV risk factor profile gradually worsened with increasing phenogroup level. Phenogroup 1 revealed the most favorable risk factors profile, with younger people and fewer participants reporting hypertension and a history of CV diseases compared to the other phenogroups. Additionally, participants assigned to phenogroup 1 achieved the highest spirometry and peak CPET values. In contrast, individuals assigned to phenogroup 5 were older and had a higher prevalence of hypertension, CV disease, and medication intake.

**Table 1:** Clinical characteristics of the training cohort (iCOMPEER) by CPET cluster

	Cluster 1 (*n* = 121)	Cluster 2 (*n* = 238)	Cluster 3 (*n* = 365)	Cluster 4 (*n* = 396)	Cluster 5 (*n* = 279)
**Anthropometrics**					

Age, y	43.19 ± 11.35	49.54 ± 11.75∗	54.65 ± 11.62∗†	60.08 ± 10.97∗†‡	64.99 ± 9.89∗†‡&
Females, n (%)	40 (33.06)	89 (37.39)	158 (43.29)∗	144 (36.36)	97 (34.77)‡
Weight, kg	92.78 ± 21.91	87.77 ± 17.24∗	82.34 ± 16.32∗†	79.63 ± 14.25∗†‡	75.32 ± 14.23∗†‡&
Height, cm	178.45 ± 8.5	174.58 ± 8.29∗	170.79 ± 8.42∗†	169.39 ± 8.39∗†‡	167.55 ± 8.18∗†‡&
Body mass index, kg/m^2^	29.11 ± 6.38	28.84 ± 5.58	28.23 ± 5.32	27.72 ± 4.38∗†	26.81 ± 4.6∗†‡&

**Medical history**					

Hypertension, n (%)	53 (43.8)	144 (60.5)∗	247 (67.67)∗	301 (76.01)∗†‡	253 (90.68)∗†‡&
DM type I or II, n (%)	7 (5.79)	22 (9.24)	41 (11.23)	57 (14.39)∗	65 (23.3)∗†‡&
Chronic kidney disease, n (%)	0 (0.0)	2 (0.84)	15 (4.11)∗†	22 (5.56)∗†	34 (12.19)∗†‡&
Asthma or COPD, n (%)	5 (4.13)	5 (2.1)	12 (3.29)	26 (6.57)†‡	18 (6.45)†
Cardiovascular disease, n (%)	37 (30.58)	112 (47.06)∗	191 (52.33)∗	273 (68.94)∗†‡	226 (81.0)∗†‡&
Cardiovascular intervention, n (%)	32 (26.45)	103 (43.28)∗	180 (49.32)∗	255 (64.39)∗†‡	216 (77.42)∗†‡&

**Medication**					

Antihypertensive drugs, n (%)	34 (28.1)	114 (47.9)∗	212 (58.08)∗†	275 (69.44)∗†‡	232 (83.15)∗†‡&
Beta blockers, n (%)	16 (13.22)	74 (31.09)∗	145 (39.73)∗†	213 (53.79)∗†‡	184 (65.95)∗†‡&
CCB, n (%)	5 (4.13)	23 (9.66)	66 (18.08)∗†	63 (15.91)∗†	68 (24.37)∗†&
ACE or ARB, n (%)	28 (23.14)	77 (32.35)	140 (38.36)∗	200 (50.51)∗†‡	174 (62.37)∗†‡&
Diuretics, n (%)	3 (2.48)	26 (10.92)∗	35 (9.59)∗	48 (12.12)∗	48 (17.2)∗†‡
Lipid lowering drugs, n (%)	36 (29.75)	110 (46.22)∗	214 (58.63)∗†	296 (74.75)∗†‡	230 (82.44)∗†‡&
Anti-thrombotic drugs, n (%)	36 (29.75)	119 (50.0)∗	202 (55.34)∗	290 (73.23)∗†‡	228 (81.72)∗†‡&
Antidiabetic drugs, n (%)	7 (5.79)	24 (10.08)	38 (10.41)	50 (12.63)∗	58 (20.79)∗†‡&

**Spirometry**					

FEV₁, L	3.84 ± 0.61	3.48 ± 0.59∗	3.11 ± 0.56∗†	2.97 ± 0.59∗†‡	2.59 ± 0.54∗†‡&
FEV₁ %predicted	103.86 ± 9.89	103.88 ± 11.88	102.63 ± 12.96	103.42 ± 14.84	98.42 ± 16.22∗†‡&
FVC₁, L	4.83 ± 0.78	4.41 ± 0.75∗	3.98 ± 0.74∗†	3.86 ± 0.74∗†‡	3.43 ± 0.67∗†‡&
FVC %predicted	107.23 ± 8.78	106.9 ± 11.48	105.78 ± 13.11	107.18 ± 14.02	102.3 ± 14.99∗†‡&
FEV₁/FVC (%)	97.02 ± 6.67	97.29 ± 5.7	97.34 ± 7.06	96.69 ± 8.1	96.39 ± 9.68

**CPET data at rest**					

HR (at rest), beats/min	73.5 ± 14.81	73.28 ± 14.05	73.88 ± 13.7	71.6 ± 13.38‡	71.46 ± 14.66‡
SBP (at rest), mm Hg	118.42 ± 18.05	122.55 ± 19.17∗	125.78 ± 20.02∗†	128.41 ± 19.75∗†	130.91 ± 21.87∗†‡
DBP (at rest), mm Hg	77.79 ± 11.62	78.77 ± 11.08	78.44 ± 11.31	77.52 ± 10.99	75.85 ± 12.79†‡
VO₂, mL/min	679.29 ± 139.06	603.55 ± 123.28∗	547.25 ± 119.58∗†	506.53 ± 113.82∗†‡	432.21 ± 104.51∗†‡&
PETO₂, mm Hg	106.04 ± 4.86	107.09 ± 5.25	107.3 ± 5.06∗	108.08 ± 5.12∗†‡	110.2 ± 5.2∗†‡&
PETCO₂, mm Hg	35.52 ± 2.79	35.08 ± 3.09	34.57 ± 3.15∗†	34.18 ± 3.17∗†	32.9 ± 3.52∗†‡&
RER	0.81 ± 0.09	0.82 ± 0.09	0.82 ± 0.08	0.83 ± 0.08	0.85 ± 0.09∗†‡&

**CPET data at peak**					

Load, watt	247.4 ± 48.02	200.76 ± 39.43∗	167.41 ± 35.45∗†	144.91 ± 32.34∗†‡	115.73 ± 30.98∗†‡&
VO₂, mL/min	2865.87 ± 530.02	2267.56 ± 419.18∗	1846.28 ± 357.03∗†	1587.16 ± 307.14∗†‡	1217.52 ± 264.8∗†‡&
VO₂ per kg, mL/kg/min	32.17 ± 8.26	26.47 ± 5.64∗	22.96 ± 4.85∗†	20.26 ± 4.07∗†‡	16.47 ± 3.78∗†‡&
VO₂ percentage predicted, %	115.95 ± 14.31	101.2 ± 13.7∗	91.11 ± 12.0∗†	82.22 ± 12.06∗†‡	68.2 ± 11.78∗†‡&
HR, bpm	171.65 ± 15.1	158.69 ± 18.93∗	148.92 ± 19.25∗†	138.55 ± 21.56∗†‡	125.11 ± 21.48∗†‡&
HR percentage predicted, %	97.16 ± 7.07	93.11 ± 9.37∗	90.16 ± 10.7∗†	86.71 ± 12.63∗†‡	80.79 ± 13.38∗†‡&
O₂ pulse, mL/beat	16.81 ± 3.36	14.53 ± 3.36∗	12.65 ± 3.11∗†	11.78 ± 3.08∗†‡	9.97 ± 2.52∗†‡&
O₂ pulse/kg, mL/beat/kg	0.19 ± 0.05	0.17 ± 0.04∗	0.16 ± 0.03∗†	0.15 ± 0.03∗†‡	0.13 ± 0.03∗†‡&
SBP, mm Hg	180.74 ± 31.95	180.72 ± 30.89	177.22 ± 30.48	175.24 ± 28.54†	169.54 ± 28.4∗†‡&
V_E_, L/min	102.6 ± 25.85	85.09 ± 21.71∗	71.7 ± 19.77∗†	64.11 ± 16.4∗†‡	52.07 ± 15.74∗†‡&
V_E_/VCO₂ slope	26.2 ± 3.21	27.26 ± 3.61∗	28.42 ± 3.97∗†	29.29 ± 4.31∗†‡	31.35 ± 4.68∗†‡&
PETO₂, mm Hg	113.57 ± 4.78	114.83 ± 4.43∗	115.02 ± 4.74∗	115.62 ± 5.1∗†	116.17 ± 4.78∗†‡
PETCO₂, mm Hg	39.7 ± 3.88	38.81 ± 3.94∗	37.87 ± 4.13∗†	37.25 ± 4.0∗†‡	35.97 ± 4.06∗†‡&
RER	1.14 ± 0.07	1.17 ± 0.07∗	1.17 ± 0.08∗	1.18 ± 0.09∗†‡	1.18 ± 0.1∗
Borg score	16.58 ± 1.55	16.26 ± 1.51	16.04 ± 1.53∗	15.79 ± 1.58∗†‡	15.36 ± 1.65∗†‡&

Data are presented as mean ± SD or number of subjects (%). Significance for between-phenogroups differences: ∗*p* < 0.05 vs. cluster 1; †*p* < 0.05 vs. cluster 2; ‡*p* < 0.05 vs. cluster 3; &*p* < 0.05 vs. cluster 4. BMI, body mass index; CPET, cardiopulmonary exercise testing; DM, diabetes mellitus; COPD, chronic obstructive pulmonary disease; CCB, calcium channel blocker; ACE, angiotensin-converting enzyme inhibitors; ARB, angiotensin receptor blocker; HR, heart rate; SBP, systolic blood pressure; DBP, diastolic blood pressure; CV, cardiovascular; FEV1, forced expiratory volume in 1 s; FVC, forced vital capacity; HR, heart rate; PETO_2_, end-tidal partial pressure of oxygen; PETCO_2_, end-tidal partial pressure of carbon dioxide; RER, respiratory exchange ratio; SBP, systolic blood pressure; VCO_2_, rate of carbon dioxide produced; V_E_, minute ventilation; V_E_/VCO_2_ slope, ventilatory efficiency; VO_2_, rate of oxygen uptake.

Tables S2 and S3 list the clinical characteristics of the participants in each phenogroup by sex. In both men and women phenogroup 1 represented the most favorable risk profile and phenogroup 5 the worst following the observations made earlier.

#### Association of CPET phenogroups with adverse events

The mean follow-up time was 4.3 years (5^th^–95^th^ percentile: 0.9 to 9.2 years). In 6,039 person-years (py) of follow-up, 297 participants experienced at least one CV event (49.2/1,000 py; 218 men, 56.5/1,000 py; 79 women, 36.3/1,000 py).

**Figure 3:**
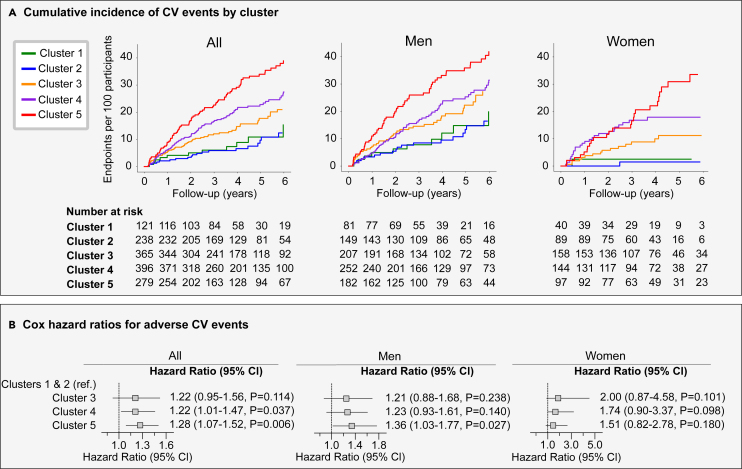
Risk for major CV events by CPET cluster derived by DTW and k-medoids (A) Shows the incidence of adverse CV events per phenogroup for all participants (left), men (middle), and women (right). (B) Presents the adjusted hazard ratios (95% Cl) for CV events against clusters 1 and 2. The models for men and women were adjusted for age, BMI, hypertension, diabetes mellitus (type I or II), CV disease, antihypertensive medication, systolic blood pressure (SBP) (at rest), HR (at rest), and peak VO2. When all participants were analyzed, sex was also included in the Cox model.

The incidence of CV events gradually increased from phenogroups 1 and 2 to 5, with event rates of 28.1/1,000 py for phenogroup 1 (14 events), 19.8/1,000 py for phenogroup 2 (21 events), 39.8/1,000 py for phenogroup 3 (63 events), 55.9/1,000 py for phenogroup 4 (98 events), and 88.1/1,000 py for phenogroup 5 (101 events) (Figure 3A). In sex-specific analysis, we observed similar trends with phenogroups 1 and 2 having the lowest and phenogroup 5 having the highest event rate in both men and women.

Figure 3B shows the adjusted hazard ratios for a CV event in phenogroups 3, 4, and 5 compared with the risk in phenogroups 1 and 2 (reference group). With men and women combined, phenogroups 4 (hazard ratio: 1.30, 95% confidence interval [CI]: 1.02–1.64, *p* = 0.033) and 5 (hazard ratio: 1.36, 95%CI: 1.08–1.72, *p* = 0.0088) had a significantly higher risk of adverse CV events compared to the reference group (clusters 1 and 2). In men, only phenogroup 5 showed a significantly higher risk (hazard ratio: 1.35; 95% CI: 1.03–1.77; *p* = 0.027), while in women we did not reach any significance. This could be attributed to the lower number of adverse events reported in women.

#### Application of the derived CPET model to the external cohort

**Figure 4:**
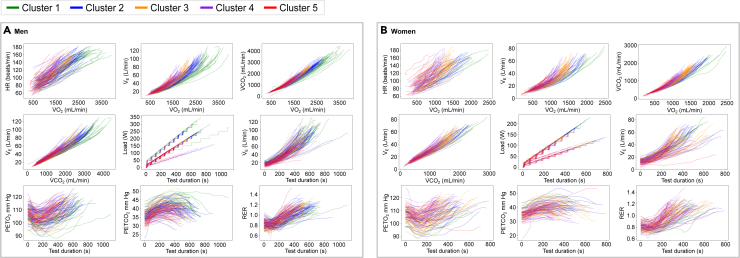
Nine-panel plot showing the clustering results of raw time-series CPET recordings derived by DTW and k-medoids for the external validation cohort (FLEMENGHO) (A and B) (A) shows the results for men and (B) shows the results for women.

The external test cohort (FLEMENGHO) comprised 266 participants of whom 44.3% were women. The mean age of the participants was 61.9 ± 8.9 years, and 53% had hypertension. After applying the already trained model to this external cohort, the derived phenogroups showed similar patterns, with individuals assigned to phenogroups 1 and 2 reaching higher peak CPET values (VO_2_, HR, VCO_2_, minute ventilation [V_E_], and load), while participants assigned to phenogroup 5 reached considerably lower peak CPET values in both sexes (Figure 4). Similarly, phenogroup 5 represented the worst clinical profile with higher prevalence of hypertension and history of CV diseases (Table 2).

**Table 2:** Clinical characteristics table of the external test cohort (FLEMENGHO) by CPET cluster

	Cluster 1 (*n* = 33)	Cluster 2 (*n* = 53)	Cluster 3 (*n* = 76)	Cluster 4 (*n* = 67)	Cluster 5 (*n* = 37)
**Anthropometrics**					

Age, y	50.58 ± 11.05	59.12 ± 9.12∗	62.51 ± 5.56∗†	66.03 ± 5.82∗†‡	67.09 ± 5.2∗†‡
Females, n (%)	8 (24.24)	16 (30.19)	45 (59.21)∗†	34 (50.75)∗†	15 (40.54)
Weight, kg	84.91 ± 19.26	86.58 ± 16.71	77.99 ± 12.46∗†	73.87 ± 12.14∗†‡	75.79 ± 17.88∗†
Height, cm	177.83 ± 8.35	175.61 ± 7.61	169.97 ± 9.0∗†	167.47 ± 8.66∗†	167.72 ± 7.27∗†
Body mass index, kg/m^2^	26.66 ± 4.75	28.08 ± 5.26	27.03 ± 4.22	26.27 ± 3.37†	26.79 ± 5.48

**Medical history**					

Hypertension, n (%)	9 (27.27)	21 (39.62)	39 (51.32)∗	45 (67.16)∗†	28 (75.68)∗†‡
DM type I or II, n (%)	1 (3.03)	6 (11.32)	6 (7.89)	10 (14.93)	8 (21.62)∗‡
Cardiovascular disease, n (%)	0 (0.0)	2 (3.77)	5 (6.58)	8 (11.94)∗	7 (18.92)∗†‡

**Medication**					

Antihypertensive drugs, n (%)	3 (9.09)	13 (24.53)	23 (30.26)∗	37 (55.22)∗†‡	26 (70.27)∗†‡
Beta blockers, n (%)	0 (0.0)	3 (5.66)	11 (14.47)∗	11 (16.42)∗	14 (37.84)∗†‡&
CCB, n (%)	0 (0.0)	5 (9.43)	10 (13.16)∗	15 (22.39)∗	8 (21.62)∗
ACE or ARB, n (%)	3 (9.09)	12 (22.64)	15 (19.74)	22 (32.84)∗	14 (37.84)∗‡
Diuretics, n (%)	1 (3.03)	8 (15.09)	7 (9.21)	14 (20.9)∗‡	9 (24.32)∗‡
Lipid lowering drugs, n (%)	6 (18.18)	15 (28.3)	20 (26.32)	25 (37.31)	15 (40.54)∗

**CPET data at rest**					

HR (at rest), beats/min	67.59 ± 10.13	69.87 ± 10.9	72.62 ± 11.92∗	74.74 ± 14.02∗†	70.91 ± 14.31
SBP (at rest), mm Hg	131.15 ± 13.12	133.3 ± 14.0	137.53 ± 17.14	134.58 ± 16.56	139.32 ± 17.89∗
DBP (at rest), mm Hg	75.29 ± 14.82	81.3 ± 9.31∗	82.33 ± 11.05∗	79.25 ± 8.86	78.12 ± 9.86
VO₂, mL/min	682.79 ± 122.53	624.83 ± 102.31∗	531.49 ± 110.45∗†	469.79 ± 108.67∗†‡	428.3 ± 135.97∗†‡
PETO₂, mm Hg	105.25 ± 4.2	108.0 ± 5.02∗	106.56 ± 4.56	109.1 ± 5.04∗‡	109.16 ± 5.39∗‡
PETCO₂, mm Hg	37.3 ± 2.93	36.11 ± 2.85	35.92 ± 3.09∗	34.18 ± 3.37∗†‡	34.25 ± 2.58∗†‡
RER	0.82 ± 0.09	0.83 ± 0.06	0.8 ± 0.07	0.84 ± 0.06‡	0.84 ± 0.08‡

**CPET data at peak**					

Load, watt	252.32 ± 38.89	213.98 ± 31.06∗	161.38 ± 26.88∗†	137.9 ± 31.87∗†‡	118.53 ± 25.14∗†‡&
VO₂, mL/min	2841.55 ± 411.06	2369.59 ± 335.41∗	1764.02 ± 295.57∗†	1501.61 ± 311.96∗†‡	1264.47 ± 248.5∗†‡&
VO₂ per kg, mL/kg/min	35.13 ± 6.16	28.51 ± 4.58∗	22.84 ± 3.59∗†	20.55 ± 3.3∗†‡	16.8 ± 4.49∗†‡&
VO₂ percentage predicted, %	120.81 ± 13.84	110.53 ± 12.02∗	99.07 ± 8.18∗†	89.68 ± 9.74∗†‡	70.85 ± 15.64∗†‡&
HR, bpm	170.05 ± 7.03	161.83 ± 14.03∗	157.45 ± 11.85∗	149.29 ± 17.44∗†‡	133.18 ± 20.12∗†‡&
HR percentage predicted, %	100.39 ± 5.87	100.42 ± 7.98	100.2 ± 7.57	97.22 ± 11.73	86.95 ± 12.8∗†‡&
O₂ pulse, mL/beat	16.72 ± 2.4	14.8 ± 2.6∗	11.29 ± 2.1∗†	10.23 ± 2.35∗†‡	9.74 ± 2.41∗†‡
O₂ pulse/kg, mL/beat/kg	0.21 ± 0.04	0.18 ± 0.03∗	0.15 ± 0.02∗†	0.14 ± 0.02∗†	0.13 ± 0.03∗†‡&
SBP, mm Hg	201.59 ± 21.39	201.04 ± 22.7	189.86 ± 31.14†	187.48 ± 23.6∗†	182.38 ± 28.39∗†
V_E_, L/min	96.24 ± 17.17	88.45 ± 16.91∗	67.62 ± 15.44∗†	59.61 ± 15.77∗†‡	47.31 ± 11.57∗†‡&
V_E_/VCO₂ slope	24.98 ± 2.1	26.64 ± 2.81∗	27.28 ± 3.26∗	124.75 ± 421.79‡	104.45 ± 354.8
PETO₂, mm Hg	112.99 ± 4.36	115.68 ± 3.94∗	114.98 ± 4.99∗	114.69 ± 5.29	111.69 ± 5.22†‡&
PETCO₂, mm Hg	42.09 ± 3.67	40.31 ± 3.36∗	39.77 ± 4.28∗	38.43 ± 3.96∗†	39.26 ± 3.96∗
RER	1.16 ± 0.06	1.18 ± 0.08	1.19 ± 0.06∗	1.18 ± 0.1	1.15 ± 0.11‡
Borg score	15.4 ± 3.0	15.23 ± 1.52	15.55 ± 1.43	12.69 ± 3.52∗†‡	14.48 ± 1.2†‡&

Data are presented as mean ± SD or number of subjects (%). Significance for between-phenogroups differences: ∗*p* < 0.05 vs. cluster 1; †*p* < 0.05 vs. cluster 2; ‡*p* < 0.05 vs. cluster 3; &*p* < 0.05 vs. cluster 4. BMI, body mass index; CPET, cardiopulmonary exercise testing; DM, diabetes mellitus; CCB, calcium channel blocker; ACE, angiotensin-converting enzyme inhibitors; ARB, angiotensin receptor blocker; HR, heart rate; SBP, systolic blood pressure; DBP, diastolic blood pressure; CV, cardiovascular; HR, heart rate; RER, respiratory exchange ratio; SBP, systolic blood pressure; VCO_2_, rate of carbon dioxide produced; V_E_, minute ventilation; V_E_/VCO_2_ slope, ventilatory efficiency; VO_2_, rate of oxygen uptake.

## Discussion

In this study, we employed a time-series clustering approach to identify distinct CRF profiles which showed clinical relevance for outcome prediction. We observed five distinct CPET patterns differing in clinical characteristics and CV risk. In specific, in both cohorts, phenogroup 1 presented the most favorable and phenogroup 5 the worst clinical profile, including CPET characteristics. Similarly, outcome analysis demonstrated that participants assigned to phenogroups 4 and 5 had a significantly higher risk of developing adverse CV events compared to phenogroups 1 and 2.

Several previously published studies highlighted the prognostic importance of CPET summary metrics.^19^^,^^20^^,^^21^ For example, Kokkinos et al.^20^ examined 667,730 US veterans with no previous evidence of heart failure or myocardial infarction. Extracting the metabolic equivalents of the participants after a standardized exercise treadmill test, they were able to distinguish 5 phenogroups associated with distinct CRF profiles. The phenogroups corresponding to the least fit individuals showed significantly higher risk of developing heart failure with preserve ejection fraction. Zannoni et al.^21^ investigated the importance of the same summary CPET variables in individuals with CV risk factors. Their analyses indicated that both peak VO_2_ and V_E_/VCO_2_ slope were significant predictors of future CV events supporting the prognostic value of CPET indexes for risk stratification. However, these studies focused primarily on the prognostic significance of the peak VO_2_ and/or the V_E_/VCO_2_ slope and did not explore the utility of other metrics recorded during CPET examination.

In our previous study,^14^ we also showed that using an unsupervised ML algorithm on the broader CPET summary metrics, we were able to separate individuals into clinically meaningful phenogroups that are associated with CV risk profiles and future CV events.

In the present study, we investigated the utility of raw CPET data beyond the summary CPET statistics (i.e., peak VO_2_ and V_E_/VCO_2_ slope) and therefore explored the usefulness of the temporal information hidden in the raw CPET data. At the same time, we were able to investigate the discriminative power of each CPET variable, providing a more detailed and comprehensible insight into the derived phenogroups. Moreover, we used an interpretability technique to highlight the most distinctive region of the time-series recordings across all clusters. Of note, the Borg score averaged between 15 and 16 in all clusters, while the RER had a high mean value of 1.14 or more on average. In our study, the Borg score was obtained after completion of CPET, which typically yields lower scores than when the patient continuously rates it during the exercise test. Therefore, considering the subjective nature of this variable, the Borg score is not a reliable index that is consistent with other studies.^22^^,^^23^

Although some previous studies investigated the temporal domain of CPET variables, they are limited in classifying symptomatic patients^16^^,^^18^ by using the available clinical diagnosis (supervised learning approach). For instance, Jablonski et al.^16^ employed a data augmentation technique to enlarge a CPET dataset recorded from 15 patients with heart failure and 15 with metabolic syndrome. After this procedure, they applied a convolutional neural network to distinguish between patients with heart failure and metabolic syndrome. Additionally, they attempted to explain the model’s decision by indicating the region of the curve that the model focused on to perform the classification. However, despite the data augmentation process, training a convolutional neural network with such a limited dataset is prone to overfitting.

One the other hand, Huang et al.^18^ presented a sparse representation-based classifier using time-series CPET data and compared it with several classification models to predict the responders in aerobic exercise intervention. From the total of 24 participants, they recorded nine metabolic indicators during CPET, namely HR, stroke volume, cardiac output, oxygen pulse, oxygen uptake per kilogram, tidal volume, ventilation volume, RER, and carbon dioxide ventilation equivalent. They noted that the limited sample size could lead to misinterpretation of the predictive performance of their proposed model.

Consequently our study provides additional knowledge to the current research as it utilizes the raw temporal CPET data to perform a multivariate time-series clustering for more personalized CRF profiling and better CV risk stratification. Furthermore, this study differs from the already reported ones due to the availability of a large patient cohort with a broad range of CPET referrals and the additional external validation cohort comprising randomly recruited individuals from the general population.

Overall, this study demonstrated the usefulness of an unsupervised ML approach to interpret the time-series CPET recordings, providing an alternative to the standard statistical analysis. Our findings suggest that the integration of the temporal characteristics of the CPET metrics may contribute to a more efficient interpretation of CPET and therefore to a more personalized evaluation of a patient’s CRF. From that perspective, the proposed approach may be a complementary tool to the current clinical practice of CPET interpretation and may guide clinicians toward more patient-tailored decision making. This could be achieved by integrating these models into commercial software as a decision support tool, potentially personalizing CV risk prediction and therefore facilitating the identification of individuals at high CV risk. This, in turn, will enable clinicians to intervene faster and more efficiently to prevent CV health from deteriorating. However, to provide conclusive evidence of the importance and clinical utility of this method in CV risk stratification, dedicated studies are still required. Further validation of the trained models using diverse populations is needed to ascertain the generalizability and clinical applicability of the developed models.

### Limitations of the study

The training sample originated from a retrospective data source, and hence it could be prone to a selection bias. Although we analyzed a heterogeneous study sample, some patient groups may be underrepresented such as individuals too sick to reach maximal CPET criteria or too healthy to be prescribed a CPET. Also, information on diseases and medication could be incomplete as medical reports remain prone to incomplete reporting. We acknowledged the relatively low number of participants included in our analysis. Nevertheless, it should be emphasized that it included more participants than other studies on ML and CPET data published so far. Furthermore, we tested the derived ML model in the independent general population cohort to evaluate its generalizability. At the same time, our outcome analysis focused mainly on incidence of CV diseases. Hence we did not include events related to pulmonary hypertension, pulmonary obstructive disease, and mitochondriopathies. Regarding the external test cohort, although it represented a different population improving the validity of the model, it comprised a lower number of participants. Our approach did not provide the discriminative power of the employed variables, but on the other hand it produced useful information about the most distinctive part in the time-series CPET data. The employed clustering algorithm (k-medoids) assumes that the data have a circular shape which might not be realistic in real-world datasets. Of note, we tested other algorithms including Gaussian mixture model which is capable of producing clusters of irregular shapes and size, but the derived clusters did not result in clinically meaningful partitioning. Finally, although the findings of this study support the usefulness of such an approach, further and extensive validation is required across different populations.

## Resource availability

### Lead contact

Further information and requests for resources should be directed to and will be fulfilled by the corresponding author, Tatiana Kuznetsova (tatiana.kouznetsova@kuleuven.be).

### Materials availability

This study did not generate new CPET data.

### Data and code availability

• All data and models reported in this paper will be shared by the corresponding author upon request.
• Python scripts are publicly available online at GitHub as of the date of the publication.
• Any additional information required to reanalyze the data reported in this paper is available from the lead contact upon request.

## Acknowledgments

The authors would like to acknowledge the guidance provided by Maria Bampa and Panagiotis Papapetrou from the Department of Computer and Systems Sciences, Faculty of Social Sciences, Stockholm University, Sweden. Their expertise in ML applications on time series was invaluable in shaping the direction of this research. This research was supported by grants from the 10.13039/501100003130Research Foundation – Flanders (Fonds Wetenschappelijk Onderzoek), Belgium (grants 1225021N, 1S07421N, and G0C5319N), and from the 10.13039/501100004497Research Council, KU Leuven (C24M/21/025, DB/22/010/BM).

## Author contributions

Conceptualization, N.C., J.C., V.C., and T.K.; data collection and curation, N.C., J.C., M.M., and T.K.; models training and validation, E.N.; software implementation, E.N.; original draft formation, E.N., N.C., and T.K.; writing – reviewing and editing of the manuscript, E.N., N.C., F.S., E.S., F.H., J.C., M.M., G.C., W.B., K.G., V.C., and T.K.; visualizations, E.N.; supervision, N.C. and T.K. All authors have read and agreed to the published version of the manuscript.

## Declaration of interests

The authors declare no competing interests.

## STAR★Methods

### Key resources table

**Table:** 

REAGENT or RESOURCE	SOURCE	IDENTIFIER
**Deposited data**		

SOURCE CODE	THIS STUDY	https://github.com/HCVE/cpet_clustering

**Software and algorithms**		

Python (version 3.9)	Python	https://www.python.org/
Dynamic time warping	TSlearn library	https://tslearn.readthedocs.io/en/stable/index.html
K-medoids	Scikit-learn-extra	https://scikit-learn-extra.readthedocs.io/en/stable/

**Other**		

Training dataset	iCOMPEER	S64901
External validation dataset	FLEMENGHO	S63118

### Experimental model and study participant details

#### Study participants

##### Training cohort

For training, we used CPET data obtained from the “*Integrative computer modelling for personalized profiling of CRF and prediction of response to ambulatory cardiac rehabilitation*” (iCOMPEER) study. The study received ethical approval from the Ethics Committee of the University of Leuven (S64901). The iCOMPEER study comprised retrospective data from 3466 individuals who underwent a maximal CPET using a cycle ergometer at the University Hospital Leuven (UZ Leuven) between 2010 and 2020. The reasons for CPET referral were broad, including screening for CV risk assessment or before participating in an exercise programme. Table S4 details the reasons for referral and the exclusion criteria applied in this study. Among other reasons, we excluded individuals who were younger than 18 years, who had a history of myocardial infarction with impact on left ventricular function (ejection fraction <50%), or who did not satisfy the maximal CPET requirement (i.e. did not reach second ventilatory threshold or did not achieve a respiratory exchange ratio above 1.05), resulting in a dataset of 2280 individuals. From those, we excluded 881 participants due to lack of time series recordings. Thus, the final dataset included raw CPET data of 1399 individuals.

Data on demographics, anthropometrics, medical history and medication intake were retrieved from the hospital’s medical repository system of the University Hospital Leuven. CPET summary metrics were retrieved from the same source.

##### External test cohort

Data from the Flemish Study on Environment, Genes and Health Outcomes (FLEMENGHO) was used to validate the performance of the trained model externally. In this study, 266 participants were randomly recruited from the general population, from northeast Belgium^24^ who recently underwent a technical examination including CPET. The Ethics Committee of the University of Leuven approved the study (S63118). Participant provided informed consent prior to participation. We employed standardized questionnaires to assess routine clinical data on demographics, medical history and lifestyle.

#### Cardiopulmonary exercise test (CPET)

In both the training and external validation cohort, participants performed CPET on a cycle ergometer (Ergometrics 800S, Ergometrics, Bitz, Germany) under the supervision of a physiotherapist and/or clinician according to the current guidelines.^25^ Three CPET devices were used over the years with two breath-by-breath analysers (Oxygen AlphaR, Jaeger, Bunnik, The Netherlands; and Med Graphics Ultima, MGC Diagnostics, Saint Paul, MN, USA). The temperature of the test room was always controlled and set at 21°C. Participants were asked to adhere to their medications prior to the examination. During CPET, a stepwise exercise protocol was applied with the target of achieving maximum exertion in 8 to 12 minutes. The incremental protocols were tailored to each individual and comprised a 20 W + 20 W/min (79.9%), 20 W + 10 W/min (12.2%), 10 W + 10 W/min (6.7%) or another incremental protocol (1.2%). During the test, the supervisor encouraged the participants to exert themselves to the maximum exertion and to retain the cycling rate between 60 and 70 revolutions per minute (rpm).

In addition, the electrocardiogram (ECG) was continuously monitored, and breath-by-breath measurements of inhaled and exhaled gas and minute ventilation were recorded throughout the examination,. Blood pressure (BP) was measured at two-minute intervals using an automated BP monitor. The test was terminated if the participant reached maximal exertion or was unable to maintain cycling rate between the desired rpm, or if any of the American Heart Association termination criteria for the CPET were met.^26^ Specifically, besides maximal exertion, the examination was terminated due to arrhythmia (n=16), angina pectoris (n=2) and orthopaedical pain (n=23). After the CPET, participants rated the maximum level of perceived exertion on the 6 to 20 Borg scale.

Peak summary metrics, including load, rate of oxygen uptake (VO_2_), rate of carbon dioxide production (VCO_2_), respiratory exchange ratio (RER), heart rate (HR), O_2_ pulse, systolic BP and minute ventilation (V_E_), were used for the statistical analysis of the derived phenogroups. We calculated the peak VO_2_ (the highest of the last three consecutive 30s-interval averages of VO_2_), the O_2_ pulse and their ratios to body weight in kilograms. Similarly, we derived the peak end-tidal partial pressure of oxygen (PETO_2_) and peak end-tidal partial pressure of carbon dioxide (PETCO_2_) by computing the highest average of the last three 30s-intervals. We also calculated the percentage-predicted HR and percentage predicted VO_2_.^27^ The $V_{E}/{VCO}_{2}$ slope was calculated through a linear regression model on the V_E_ and VCO_2_ recordings until the respiratory compensation point was reached (VT2).^28^ The second ventilatory threshold (VT2) was defined by adopting the ventilatory equivalent $V_{E}/{VCO}_{2}$ method as defined elsewhere.^29^ Table S5 lists the formulas for the calculation of the CPET summary indexes.

#### Other clinical measurements

From the training cohort, we also retrieved summary indexes of spirometry recordings, including the forced expiratory volume in 1 second (FEV_1_), the forced vital capacity (FVC), their ratio and the percentage predicted FEV_1_ and FVC by taking into account the participant’s age, sex and height.^30^

Hypertension was defined as a systolic BP higher than 140 mmHg and/or a diastolic BP above 90 mmHg and/or the intake of antihypertensive drugs. Diabetes mellitus was defined by self-report, medical report, a fasting serum glucose level above 7 mmol/L and/or the intake of antidiabetic medication.

#### Outcome assessment

In the iCOMPEER cohort, we collected information on adverse CV events. From the medical repository system of UZ Leuven we collected fatal and non-fatal CV events until June 2023. Events included coronary events, symptomatic heart failure, valvular heart disease requiring surgical intervention, heart block, pacemaker implantation, atrial fibrillation, stroke, transient ischemic attacks, aortic aneurysm, pulmonary heart disease, pulmonary embolism or infarction, peripheral vascular disease, including arterial embolism or thrombosis. As highlighted in our previous work^14^ a 3-months period for a first CV event was respected to exclude events resulting from residual therapy (e.g., planned percutaneous coronary intervention for residual lesions).

### Method details

Figure S2 presents the computational pipeline developed to identify CPET phenogroups through unsupervised ML. We used a Python 3.9 environment with common Python libraries for signal processing (SciPy,^31^ NumPy^32^) and for model training (tslearn^33^ and scikit-learn^34^). All Python scripts created for this analysis are publicly available in https://github.com/HCVE/cpet_clustering.

#### Signal pre-processing

Excessive movement during CPET or temporarily inappropriate sealing of the mask may have caused noise or errors in the breath-by-breath measurements. Therefore, before training the model, we employed signal processing techniques to correct erroneous measurements and reduce the noise in the CPET recordings. For this, we used simple statistics to identify regions within the time series curves with inaccurate measurements due to errors or noise, and a moving average filter to correct them.

In detail, we first replaced invalid characters (i.e. “-” or “>>”) with 0s to achieve fully numerical sequences. However, the insertion of zero values resulted in physiologically non valid curves. Hence we further corrected these values by applying the following formula.

$${corrected\;value}_{t}={measurement}_{t-1}+0.5\times \left|{measurement}_{t-1}-{measurements}_{t-2}\right|$$

where *measurement*_*t*_ indicates the value of the breath-by-breath gas exchange recordings at a specific time-stamp.

Next, we ensured that the load during the test was monotonically increasing and that the duration of the test was sufficiently long enough (more than 10 samples). These first two steps identified and corrected potential instrumentation errors such as inadequate sealing of the mask for the breath-by-breath recordings.

To reduce noise originating from excessive movement during the CPET we first applied a filter based on local statistics and then a moving average filter, to identify and correct measurements that corresponded to extreme spikes or dips.

During the first step we assessed if a measurement fell within the μ±σ range, where μ is the average value of the two measurements before and two measurement after the sample under investigation and σ their standard deviation. Values exceeding the μ±σ range were replaced by the average value of the previous and next measurement. Finally, if the recording was long enough (i.e. at least 30 samples) we applied the moving average filter with a window of 11 samples or we applied for a second time the first step to further refine the time series recordings.

#### Model training

We applied dynamic time warping (DTW) combined with k-medoids algorithm to derive CPET phenogroups (clusters). DTW calculates the distance between two time series data. We applied DTW because *(i)* it can handle data of different length, *(ii)* it is suitable for multivariate data analysis and *(iii)* it performs a temporal alignment, meaning it can capture and compare the shape of the time series.^35^

K-medoids is a widely used algorithm to perform clustering tasks.^36^ It is similar to k-means as they both rely on the Euclidean distance and both provide the centre of each cluster. However, in k-medoids, the centre corresponds to a real recording that already exists in the dataset, making this approach more robust to noise and outliers. Because of substantial sex differences in CPET test results, we constructed one clustering model for men and another for women.

From the temporal CPET recordings, we used the tracings of HR, VO_2_, RER, PETO_2_ and PETCO_2_ for clustering. We selected these variable tracings after investigating the cross-correlation in the temporal domain but also after taking into consideration the correlation of the peak summary metrics of the most used CPET variables including load, ventilation ($\dot{V}$E) and exhaled carbon dioxide ($\dot{V}$CO_2_).

To investigate the correlation between time series data we first evaluated the cross-correlation with respect to the time lag of time series curves in the dataset for each CPET variable separately. In this way, we constructed an 8-by-8 grid comparing the temporal cross-correlation of each feature (Figure S3). Of note, all variables appear to have higher correlation for small or no temporal lag indicating no temporal shift. Among the gas exchange variables ($\dot{V}$O_2_, $\dot{V}$CO_2_, $\dot{V}$E, RER, PETO_2_, PETCO_2_) we observed a higher correlation between $\dot{V}$O_2_, $\dot{V}$CO_2_, $\dot{V}$E at small time-lags (or at zero time lag) and between RER and $\dot{V}$CO_2_ for zero or small time lags. Additionally, we observed a negative correlation between PETO_2_ and PETCO_2_. As mentioned earlier for all combinations we observed a higher correlation only for small time lags (mostly at zero time lag) suggesting that we could investigate the raw time series without taking into consideration any potential time shift.

Furthermore, we constructed the cross-correlation matrix using Spearman’s correlation (Figure S4). Similarly, we observed higher correlations between $\dot{V}$O_2_, $\dot{V}$CO_2_, and $\dot{V}$E.

It should be highlighted that both temporal cross-correlation and Spearman’s correlation require stationary data. Therefore, throughout this analysis we transformed the time-series recordings into stationary signals. CPET recordings are non-stationary since all variables exhibit a specific trend as the duration of the test increases. For example, $\dot{V}$O_2_, $\dot{V}$CO_2_, $\dot{V}R$E are increasing as CPET progresses in time. To overcome this limitation we first applied Augmented Dickey-Fuller to test if indeed the time series recordings of each individual were stationary or not. If the test showed non-stationarity, we calculated the derivative. Then we applied again the Augmented Dickey-Fuller to test for stationarity. This process was repeated until all CPET recordings were stationary.

Our analysis suggested that the selected variables were the least correlated between the most used CPET variables. On the other hand, VCO_2_ and load tracings were not included as they are highly correlated with VO_2_ both in the temporal domain and the peak summary metrics (Figures S3 and S4).^14^ To find the optimal number of clusters, we combined the silhouette score and the Dunn index to calculate and evaluate the cluster validity index (CVI) as: $cvi=\sqrt{silhouette\times dunn}$.^37^ Finally, we applied the trained models on the raw CPET recordings of the external validation cohort (FLEMENGHO), providing CPET phenogroups for men and women separately.

#### Interpretability of the trained model

To better understand the clustering results and explain the trained ML model, we further investigated which part of the time series curves impacted the model’s decision the most. To achieve this, we first separated the dataset into two separate subsets; the first included the curves assigned to the cluster under investigation (subset 1) and the second the curves that were not assigned to that cluster (subset 2). Then, for each recording in subset 1 we calculated the per-sample dynamic time warping distance with respect to the recordings in subset 2. From those, we only retained those that corresponded to the top q^th^ quantile (here set to the 50th quantile).

In this way, we obtained the time stamps of the samples that were the most distinctive for the recordings in subset 1 as compared to those in subset 2. Then, we isolated the time stamps that occurred more frequently based on a user-defined threshold (set at 50% of the dataset). Finally, from the most frequent time stamps we only kept those that formed a continuous region. This procedure was iterated until all clusters were used as the group under investigation.

### Quantification and statistical analysis

To perform statistical analysis and database management we employed SAS software, version 9.4 (SAS Institute, Cary, NC, USA) and Python environment 3.9 (https://www.python.org). We used the standard normal (Z) and chi-square distributions to calculate the mean values of continuous variables and the proportions of the categorical variables, respectively. To evaluate the clinical importance of the CPET phenogroups, the clinical and summary indexes of CPET characteristics of the phenogroups in both iCOMPEER and FLEMENGHO cohorts were compared. In the iCOMPEER cohort, we applied the Kaplan-Meier method to assess the cumulative incidence of adverse CV events per CPET phenogroup in all participants and by sex. Finally, we used Cox regression to calculate standardized hazard ratios (HR) for incident CV events. We adjusted hazard ratios for important covariables including age, sex, body mass index, hypertension, history of diabetes mellitus and/or CV disease, intake of antihypertensive medication, systolic BP (at rest), HR (at rest) and peak VO_2_. In the study participants who experienced CV events, we only considered the first event per participant.

### Additional resources

This study utilizes CPET recordings that were obtained during the “*Integrative computer modelling for personalized profiling of CRF and prediction of response to ambulatory cardiac rehabilitation*” (iCOMPEER) study (S64901) and the Flemish Study on Environment, Genes and Health Outcomes (FLEMENGHO - S63118).
