# Supplementary material for: Improving cardiovascular risk stratification through multivariate time-series analysis of cardiopulmonary exercise test data
Source: iScience. 2024 Aug 22;27(9):110792. doi: 10.1016/j.isci.2024.110792 (PMC11403400; doi:10.1016/j.isci.2024.110792)

iScience, Volume *27*

Supplemental information

Improving cardiovascular risk stratification through multivariate time-series analysis of cardiopulmonary exercise test data

Evangelos Ntalianis, Nicholas Cauwenberghs, František Sabovčik, Everton Santana, Francois Haddad, Jomme Claes, Matthijs Michielsen, Guido Claessen, Werner Budts, Kaatje Goetschalckx, Véronique Cornelissen, and Tatiana Kuznetsova

SUPPLEMENTAL INFORMATION

Supplemental Table *S1*: Clinical characteristics of the 1399 participants by sex in the training sample (iCOMPEER)

	Men (n=871)	Women (n=528)	P Value
**Anthropometrics**			
Age, y	58.79 ± 11.83	52.42 ± 13.54	<0.0001
Weight, Kg	86.49 ± 16.0	74.6 ± 15.7	<0.0001
Height, cm	175.3 ± 7.14	164.05 ± 6.96	<0.0001
Body mass index, kg/m²	28.12 ± 4.7	27.76 ± 5.79	<0.0001
**Medical history**			
Hypertension, n(%)	704 (80.83)	294 (55.68)	<0.0001
DM type I or II, n(%)	146 (16.76)	46 (8.71)	<0.0001
Chronic kidney disease, n(%)	50 (5.74)	23 (4.36)	0.0006
Asthma or COPD, n(%)	40 (4.59)	26 (4.92)	0.98
Cardiovascular disease, n(%)	655 (75.2)	184 (34.85)	<0.0001
Cardiovascular intervention, n(%)	630 (72.33)	156 (29.55)	<0.0001
**Medication**			
Antihypertensive drugs, n(%)	623 (71.53)	244 (46.21)	<0.0001
Lipid lowering drugs, n(%)	679 (77.96)	207 (39.2)	<0.0001
Anti-thrombotic drugs, n(%)	674 (77.38)	201 (38.07)	<0.0001
Anti-diabetic drugs, n(%)	128 (14.7)	49 (9.28)	<0.0001
Beta blockers, n(%)	442 (50.75)	190 (35.98)	<0.0001
CCB, n(%)	154 (17.68)	71 (13.45)	<0.0001
ACE or ARB, n(%)	468 (53.73)	151 (28.6)	<0.0001
Diuretics, n(%)	98 (11.25)	62 (11.74)	0.056
**Spirometry**			
FEV_1, L	3.4 ± 0.61	2.56 ± 0.45	<0.0001
FEV_1 %predicted	102.45 ± 13.9	102.22 ± 13.93	0.40
FVC_1, L	4.39 ± 0.73	3.26 ± 0.55	<0.0001
FVC %predicted	104.5 ± 12.68	108.22 ± 14.04	<0.0001
FEV_1/FVC (%)	98.18 ± 7.85	94.71 ± 7.18	<0.0001
**CPET data at rest**			
HR (at rest), beats/min	69.82 ± 12.87	77.23 ± 14.57	<0.0001
SBP (at rest), mm Hg	129.14 ± 19.71	121.78 ± 20.57	<0.0001
DBP (at rest), mm Hg	78.23 ± 11.64	76.73 ± 11.38	0.001
$\dot{V}O_2$, mL/min	575.66 ± 132.1	464.69 ± 115.68*	<0.0001
$PETO_2$, mm Hg	108.03 ± 5.24	107.83 ± 5.31	0.48
$PETCO_2$, mm Hg	34.44 ± 3.27	34.05 ± 3.31*	0.032
RER	0.84 ± 0.08	0.81 ± 0.08*	<0.0001
**CPET data at peak**			
Load, watt	184.95 ± 47.44	127.65 ± 36.69	<0.0001
$\dot{V}O_2$, ml/min	2052.79 ± 558.08	1402.58 ± 368.76	<0.0001
$\dot{V}O_2$ per kg, ml/kg/min	24.12 ± 6.68	19.29 ± 5.44	<0.0001
$\dot{V}O_2$ percentage predicted, %	88.97 ± 19.19	86.11 ± 17.23	0.017
HR, bpm	142.93 ± 23.25	148.05 ± 25.54	<0.0001
HR percentage predicted, %	88.59 ± 12.4	88.15 ± 12.37	0.513
O_2 pulse, ml/beat	14.38 ± 3.1	9.53 ± 2.05	<0.0001

O_2 pulse/kg, ml/beat/kg	0.17 ± 0.03	0.13 ± 0.03	<0.0001
SBP, mm Hg	183.86 ± 28.18	163.11 ± 28.35	<0.0001
$\dot{V}E$, L/min	81.13 ± 22.51	53.2 ± 13.78	<0.0001
$\dot{V}E/\dot{V}CO_2$ slope	29.02 ± 4.33	28.59 ± 4.46	0.0059
$PETO_2$, mm Hg	115.42 ± 4.95	115.01 ± 4.68	0.12
$PETCO_2$, mm Hg	37.63 ± 4.21	37.64 ± 4.12	0.97
RER	1.18 ± 0.08	1.16 ± 0.09	<0.0001
Borg score	15.85 ± 1.56	16.03 ± 1.68	0.148

Data are presented as mean ± SD or number of subjects (%). BMI, body mass index; CPET, cardiopulmonary exercise testing; DM, Diabetes mellitus; COPD, chronic obstructive pulmonary disease; CCB, Calcium channel blocker; ACE, angiotensin-converting enzyme inhibitors; ARB, angiotensin receptor blocker; HR, heart rate; SBP, systolic blood pressure; DBP, diastolic blood pressure; CV, cardiovascular; FEV1, forced expiratory volume in 1 s; FVC, forced vital capacity; HR, heart rate; RER, respiratory exchange ratio; SBP, systolic blood pressure; $\dot{V}CO_2$, rate of carbon dioxide produced; $\dot{V}E$, minute ventilation; $\dot{V}E/\dot{V}CO_2$ slope, ventilatory efficiency; $\dot{V}O_2$, rate of oxygen uptake.

Supplemental Table S2: Clinical characteristics of men in the training cohort (iCOMPEER) per cluster.

	Cluster 1 (n=81)	Cluster 2 (n=149)	Cluster 3 (n=207)	Cluster 4 (n=252)	Cluster 5 (n=182)
**Anthropometrics**					
Age, y	45.79 ± 10.59	52.04 ± 11.62*	58.25 ± 9.75*†	62.14 ± 9.96*†‡	66.1 ± 9.06*†‡&
Weight, kg	96.6 ± 22.88	91.55 ± 16.64	87.88 ± 13.82*†	84.33 ± 12.98*†‡	79.27 ± 13.67*†‡&
Height, cm	182.3 ± 6.36	178.3 ± 6.86*	175.89 ± 6.27*†	173.65 ± 6.27*†‡	171.35 ± 6.28*†‡&
Body mass index, kg/m²	29.03 ± 6.51	28.8 ± 4.93	28.42 ± 4.36	27.98 ± 4.19	26.98 ± 4.33*†‡&
**Medical history**					
Hypertension, n(%)	46 (56.79)	109 (73.15)*	168 (81.16)*	211 (83.73)*†	170 (93.41)*†‡&
DM type I or II, n(%)	4 (4.94)	20 (13.42)*	28 (13.53)*	44 (17.46)*	50 (27.47)*†‡&
Chronic kidney disease, n(%)	0 (0.0)	2 (1.34)	8 (3.86)	16 (6.35)*†	24 (13.19)*†‡&
Asthma or COPD, n(%)	5 (6.17)	3 (2.01)	4 (1.93)	14 (5.56)‡	14 (7.69) †‡
Cardiovascular disease, n(%)	35 (43.21)	100 (67.11)*	148 (71.5)*	212 (84.13)*†‡	160 (87.91)*†‡
Cardiovascular intervention, n(%)	32 (39.51)	96 (64.43)*	144 (69.57)*	202 (80.16)*†‡	156 (85.71)*†‡
**Medication**					
Antihypertensive drugs, n(%)	30 (37.04)	88 (59.06)*	148 (71.5)*†	199 (78.97)*†	158 (86.81)*†‡&
Lipid lowering drugs, n(%)	36 (44.44)	96 (64.43)*	163 (78.74)*†	220 (87.3)*†‡	164 (90.11)*†‡
Anti-thrombotic drugs, n(%)	35 (43.21)	106 (71.14)*	154 (74.4)*	216 (85.71)*†‡	163 (89.56)*†‡
Anti-diabetic drugs, n(%)	4 (4.94)	20 (13.42)*	23 (11.11)	36 (14.29)*	45 (24.73)*†‡&
Beta blockers, n(%)	14 (17.28)	61 (40.94)*	96 (46.38)*	150 (59.52)*†‡	121 (66.48)*†‡
CCB, n(%)	4 (4.94)	19 (12.75)	46 (22.22)*†	40 (15.87)*	45 (24.73)*†&
ACE or ARB, n(%)	26 (32.1)	61 (40.94)	106 (51.21)*	153 (60.71)*†‡	122 (67.03)*†‡
Diuretics, n(%)	2 (2.47)	16 (10.74)*	20 (9.66)*	29 (11.51)*	31 (17.03)*‡
**Spirometry**					
FEV_1, L	4.17 ± 0.52	3.82 ± 0.5*	3.44 ± 0.48*†	3.26 ± 0.54*†‡	2.84 ± 0.52*†‡&
FEV_1 %predicted	104.7 ± 10.46	106.26 ± 11.39	103.27 ± 12.63†	102.81 ± 15.1†	96.79 ± 15.54*†‡&
FVC_1, L	5.29 ± 0.58	4.87 ± 0.61*	4.43 ± 0.61*†	4.26 ± 0.64*†‡	3.74 ± 0.63*†‡&
FVC %predicted	107.41 ± 8.38	108.07 ± 11.66	104.74 ± 12.07†	105.29 ± 13.21†	98.83 ± 13.7*†‡&
FEV_1/FVC (%)	97.51 ± 6.56	98.55 ± 5.51	98.82 ± 7.02	97.72 ± 8.63	98.07 ± 9.55
**CPET data at rest**					
HR (at rest), beats/min	71.4 ± 13.35	69.18 ± 12.38	70.43 ± 12.07	68.99 ± 12.46	70.09 ± 14.3

SBP (at rest), mm Hg	122.07 ± 18.5	126.45 ± 18.89	130.27 ± 19.87*	130.92 ± 19.1*†	130.73 ± 20.58*
DBP (at rest), mm Hg	79.52 ± 11.63	79.35 ± 10.94	80.29 ± 11.16	77.46 ± 11.24‡	75.45 ± 12.61*†‡
$\dot{V}O_2$, mL/min	722.65 ± 131.22	635.73 ± 111.6*	602.15 ± 108.8*†	552.43 ± 103.21*†‡	463.09 ± 103.21*†‡&
$PETO_2$, mm Hg	105.86 ± 4.82	106.79 ± 5.31	107.34 ± 5.12*	108.25 ± 4.85*†	110.51 ± 5.08*†‡&
$PETCO_2$, mm Hg	35.92 ± 2.69	35.55 ± 3.03	34.78 ± 3.07*†	34.2 ± 3.15*†‡	32.85 ± 3.37*†‡&
RER	0.82 ± 0.09	0.83 ± 0.08	0.84 ± 0.09	0.84 ± 0.08	0.86 ± 0.08*†‡&
**CPET data at peak**					
Load, watt	273.27 ± 34.71	224.93 ± 25.05*	192.78 ± 19.86*†	164.96 ± 19.32*†‡	131.7 ± 22.75*†‡&
$\dot{V}O_2$, ml/min	3192.86 ± 285.15	2564.76 ± 184.18*	2142.6 ± 116.59*†	1804.13 ± 118.46*†‡	1368.43 ± 188.41*†‡&
$\dot{V}O_2$ per kg, ml/kg/min	34.7 ± 8.0	28.79 ± 4.86*	24.96 ± 3.99*†	21.89 ± 3.6*†‡	17.71 ± 3.69*†‡&
$\dot{V}O_2$ percentage predicted, %	116.22 ± 15.21	102.34 ± 15.16*	92.56 ± 12.01*†	83.83 ± 12.7*†‡	68.95 ± 12.5*†‡&
HR, bpm	170.79 ± 15.33	156.34 ± 18.0*	146.11 ± 17.97*†	136.19 ± 20.32*†‡	125.28 ± 19.98*†‡&
HR percentage predicted, %	98.07 ± 7.09	93.14 ± 9.26*	90.5 ± 11.19*†	86.42 ± 12.72*†‡	81.48 ± 12.6*†‡&
O_2 pulse, ml/beat	18.8 ± 2.04	16.63 ± 2.27*	14.88 ± 2.0*†	13.55 ± 2.21*†‡	11.14 ± 2.04*†‡&
O_2 pulse/kg, ml/beat/kg	0.2 ± 0.04	0.19 ± 0.03*	0.17 ± 0.03*†	0.16 ± 0.03*†‡	0.14 ± 0.03*†‡&
SBP, mm Hg	192.73 ± 29.66	190.26 ± 28.43	187.18 ± 27.4	182.0 ± 25.5*†‡	173.46 ± 28.27*†‡&
$\dot{V}E$, L/min	115.54 ± 20.61	97.57 ± 16.66*	84.72 ± 15.33*†	73.04 ± 12.75*†‡	59.46 ± 13.91*†‡&
$\dot{V}E/\dot{V}CO_2$ slope	26.11 ± 2.93	27.5 ± 3.59*	28.59 ± 3.68*†	29.5 ± 4.21*†‡	31.39 ± 4.86*†‡&
$PETO_2$, mm Hg	113.83 ± 4.7	114.93 ± 4.48	115.29 ± 4.96*	115.5 ± 5.16*	116.57 ± 4.85*†‡&
$PETCO_2$, mm Hg	39.64 ± 3.87	38.87 ± 4.04	37.89 ± 4.11*†	37.27 ± 4.03*†	35.94 ± 4.09*†‡&
RER	1.15 ± 0.07	1.17 ± 0.07*	1.17 ± 0.08*	1.18 ± 0.08*	1.2 ± 0.1*†‡
Borg score	16.62 ± 1.32	16.15 ± 1.48*	15.84 ± 1.47*	15.81 ± 1.51*†	15.34 ± 1.69*†‡&

Data are presented as mean ± SD or number of subjects (%). Significance for between-phenogroups differences: *P<0.05 vs Cluster 1; †P<0.05 vs Cluster 2; ‡P<0.05 vs Cluster 3; &P<0.05 vs Cluster 4. BMI, body mass index; CPET, cardiopulmonary exercise testing; DM, Diabetes mellitus; COPD, chronic obstructive pulmonary disease; CCB, Calcium channel blocker; ACE, angiotensin-converting enzyme inhibitors; ARB, angiotensin receptor blocker; HR, heart rate; SBP, systolic blood pressure; DBP, diastolic blood pressure; CV, cardiovascular; FEV1, forced expiratory volume in 1 s; FVC, forced vital capacity; HR, heart rate; $PETO_2$, end-tidal partial pressure of oxygen; $PETCO_2$, end-tidal partial pressure of carbon dioxide; RER, respiratory exchange ratio; SBP, systolic blood pressure; $\dot{V}CO_2$, rate of carbon dioxide produced; $\dot{V}E$, minute ventilation; $\dot{V}E/\dot{V}CO_2$ slope, ventilatory efficiency; $\dot{V}O_2$, rate of oxygen uptake.

Supplemental Table S3: Clinical characteristics of women in the training cohort (iCOMPEER) per cluster.

	Cluster 1 (n=40)	Cluster 2 (n=89)	Cluster 3 (n=158)	Cluster 4 (n=144)	Cluster 5 (n=97)
**Anthropometrics**					
Age, y	37.94 ± 11.03	45.34 ± 10.73*	49.95 ± 12.17*†	56.48 ± 11.71*†‡	62.89 ± 10.98*†‡&
Weight, kg	85.03 ± 17.39	81.46 ± 16.36	75.09 ± 16.5*†	71.43 ± 12.54*†‡	67.92 ± 12.14*†‡&
Height, cm	170.65 ± 6.78	168.37 ± 6.56	164.1 ± 5.76*†	161.93 ± 6.13*†‡	160.42 ± 6.37*†‡
Body mass index, kg/m²	29.29 ± 6.1	28.91 ± 6.51	27.97 ± 6.35	27.26 ± 4.68*†	26.49 ± 5.05*†
**Medical history**					
Hypertension, n(%)	7 (17.5)	35 (39.33)*	79 (50.0)*	90 (62.5)*†‡	83 (85.57)*†‡&
DM type I or II, n(%)	3 (7.5)	2 (2.25)	13 (8.23)	13 (9.03)†	15 (15.46)†
Chronic kidney disease, n(%)	0 (0.0)	0 (0.0)	7 (4.43)†	6 (4.17)	10 (10.31)*†
Asthma or COPD, n(%)	0 (0.0)	2 (2.25)	8 (5.06)	12 (8.33)	4 (4.12)
Cardiovascular disease, n(%)	2 (5.0)	12 (13.48)	43 (27.22)*†	61 (42.36)*†‡	66 (68.04)*†‡&
Cardiovascular intervention, n(%)	0 (0.0)	7 (7.87)	36 (22.78)*†	53 (36.81)*†‡	60 (61.86)*†‡&
**Medication**					
Antihypertensive drugs, n(%)	4 (10.0)	26 (29.21)*	64 (40.51)*	76 (52.78)*†‡	74 (76.29)*†‡&
Lipid lowering drugs, n(%)	0 (0.0)	14 (15.73)*	51 (32.28)*†	76 (52.78)*†‡	66 (68.04)*†‡&
Anti-thrombotic drugs, n(%)	1 (2.5)	13 (14.61)*	48 (30.38)*†	74 (51.39)*†‡	65 (67.01)*†‡&
Anti-diabetic drugs, n(%)	3 (7.5)	4 (4.49)	15 (9.49)	14 (9.72)	13 (13.4)†
Beta blockers, n(%)	2 (5.0)	13 (14.61)	49 (31.01)*†	63 (43.75)*†‡	63 (64.95)*†‡&
CCB, n(%)	1 (2.5)	4 (4.49)	20 (12.66)†	23 (15.97)*†	23 (23.71)*†‡
ACE or ARB, n(%)	2 (5.0)	16 (17.98)*	34 (21.52)*	47 (32.64)*†‡	52 (53.61)*†‡&
Diuretics, n(%)	1 (2.5)	10 (11.24)	15 (9.49)	19 (13.19)	17 (17.53)*
**Spirometry**					
FEV_1, L	3.17 ± 0.37	2.87 ± 0.39*	2.61 ± 0.39*†	2.44 ± 0.38*†‡	2.07 ± 0.33*†‡&
FEV_1 %predicted	102.14 ± 8.45	99.57 ± 11.92	101.68 ± 13.35	104.5 ± 14.31†	101.91 ± 17.11
FVC_1, L	3.9 ± 0.53	3.57 ± 0.47*	3.31 ± 0.53*†	3.14 ± 0.51*†‡	2.76 ± 0.36*†‡&
FVC %predicted	106.84 ± 9.53	104.78 ± 10.97	107.35 ± 14.27	110.59 ± 14.93†	109.73 ± 15.7†
FEV_1/FVC (%)	96.04 ± 6.81	95.01 ± 5.59	95.14 ± 6.73	94.84 ± 6.81	92.82 ± 9.36‡
**CPET data at rest**					
HR (at rest), beats/min	77.75 ± 16.59	80.15 ± 14.01	78.41 ± 14.38	76.15 ± 13.69†	74.02 ± 14.98†‡

SBP (at rest), mm Hg	111.02 ± 14.54	116.02 ± 17.81	119.9 ± 18.64*	124.01 ± 20.08*†	131.25 ± 24.09*†‡&
DBP (at rest), mm Hg	74.3 ± 10.79	77.8 ± 11.24	76.01 ± 11.06	77.62 ± 10.52	76.6 ± 13.1
VO_2, mL/min	591.48 ± 110.03	549.66 ± 123.1	475.32 ± 91.67*†	426.2 ± 82.73*†‡	374.28 ± 79.29*†‡&
$PETO_2$, mm Hg	106.4 ± 4.93	107.6 ± 5.12	107.25 ± 4.97	107.79 ± 5.54	109.64 ± 5.37*†‡&
$PETCO_2$, mm Hg	34.7 ± 2.8	34.31 ± 3.04	34.3 ± 3.22	34.16 ± 3.21	33.0 ± 3.77*†‡&
RER	0.79 ± 0.09	0.8 ± 0.08	0.8 ± 0.07	0.81 ± 0.08	0.84 ± 0.09*†‡&
**CPET data at peak**					
Load, watt	195.0 ± 20.86	160.28 ± 22.16*	134.18 ± 20.95*†	109.83 ± 16.98*†‡	85.77 ± 20.34*†‡&
$\dot{V}O_2$, ml/min	2203.7 ± 173.59	1770.01 ± 132.92*	1458.06 ± 104.47*†	1207.46 ± 91.15*†‡	934.37 ± 110.37*†‡&
$\dot{V}O_2$ per kg, ml/kg/min	27.05 ± 6.13	22.57 ± 4.63*	20.35 ± 4.65*†	17.4 ± 3.18*†‡	14.13 ± 2.66*†‡&
$\dot{V}O_2$ percentage predicted, %	115.41 ± 12.29	99.3 ± 10.55*	89.22 ± 11.73*†	79.4 ± 10.25*†‡	66.8 ± 10.13*†‡&
HR, bpm	173.4 ± 14.47	162.63 ± 19.78*	152.6 ± 20.22*†	142.67 ± 22.99*†‡	124.8 ± 24.04*†‡&
HR percentage predicted, %	95.31 ± 6.64	93.06 ± 9.54	89.72 ± 10.01*†	87.21 ± 12.45*†	79.51 ± 14.66*†‡&
O_2 pulse, ml/beat	12.77 ± 1.15	11.02 ± 1.4*	9.72 ± 1.42*†	8.69 ± 1.6*†‡	7.76 ± 1.71*†‡&
O_2 pulse/kg, ml/beat/kg	0.16 ± 0.03	0.14 ± 0.03*	0.13 ± 0.03*	0.12 ± 0.02*†‡	0.12 ± 0.03*†‡&
SBP, mm Hg	156.45 ± 20.62	164.74 ± 28.11	164.18 ± 29.38	163.4 ± 29.69	162.2 ± 27.15
$\dot{V}E$, L/min	76.39 ± 11.62	64.18 ± 9.92*	54.64 ± 9.03*†	48.48 ± 8.45*†‡	38.22 ± 7.46*†‡&
$\dot{V}E/\dot{V}CO_2$ slope	26.38 ± 3.71	26.85 ± 3.61	28.2 ± 4.31*†	28.92 ± 4.45*†	31.26 ± 4.33*†‡&
$PETO_2$, mm Hg	113.06 ± 4.91	114.68 ± 4.33	114.68 ± 4.41*	115.83 ± 4.98*‡	115.42 ± 4.54*
$PETCO_2$, mm Hg	39.84 ± 3.88	38.72 ± 3.76	37.85 ± 4.16*	37.22 ± 3.94*†	36.02 ± 4.0*†‡&
RER	1.14 ± 0.06	1.16 ± 0.07	1.16 ± 0.08	1.18 ± 0.1*‡	1.15 ± 0.09&
Borg score	16.48 ± 1.94	16.45 ± 1.56	16.29 ± 1.57	15.77 ± 1.69*†‡	15.4 ± 1.56*†‡

Data are presented as mean ± SD or number of subjects (%). Significance for between-phenogroups differences: *P<0.05 vs Cluster 1; †P<0.05 vs Cluster 2; ‡P<0.05 vs Cluster 3; &P<0.05 vs Cluster 4. BMI, body mass index; CPET, cardiopulmonary exercise testing; DM, Diabetes mellitus; COPD, chronic obstructive pulmonary disease; CCB, Calcium channel blocker; ACE, angiotensin-converting enzyme inhibitors; ARB, angiotensin receptor blocker; HR, heart rate; SBP, systolic blood pressure; DBP, diastolic blood pressure; CV, cardiovascular; FEV1, forced expiratory volume in 1 s; FVC, forced vital capacity; HR, heart rate; $PETO_2$, end-tidal partial pressure of oxygen; $PETCO_2$, end-tidal partial pressure of carbon dioxide; RER, respiratory exchange ratio; SBP, systolic blood pressure; VCO_2, rate of carbon dioxide produced; $\dot{V}E$, minute ventilation; $\dot{V}E/\dot{V}CO2$ slope, ventilatory efficiency; $\dot{V}O_2$, rate of oxygen uptake.

Supplemental Table S*4*: Reasons for CPET referral and the exclusion criteria in the training cohort (iCOMPEER).

**Reasons for referral**	Recent revascularisation including PCI<br>CV risk assessment in hypertension, obesity and/or diabetes mellitus<br>Screening before participating in an exercise programme<br>Differential diagnosis of dyspnoea, chronic fatigue syndrome, other exercise-limiting condition
**Exclusion criteria**	Age < 18 and age > 80<br>Previous myocardial infarction with left ventricular ejection fraction < 50%<br>Symptomatic heart failure, congenital heart disease, cardiomyopathy, cardiac surgery, artificial pacemaker<br>Auto-immune disease, drug abuse, pregnancy<br>Non-compliance to maximal CPET criteria (VT_2 not reached and/or RER<1.05)

Supplemental Table S*5*: Formulas used to calculate peak CPET summary metrics

**Summary CPET metrics at peak**	**Formula**
O_2 pulse (ml/beat)	$\frac{VO_{2\ (peak)}}{HR_{(peak)}}$
HR percentage predicted	$100 \times \frac{HR}{220 - age}$
Predicted VO_2[1]	$men{:} -69 + 1.48 \times age + 14.02 \times height + 7.44 \times weight - 0.2256 \times age^2$<br>$women{:}\ -588 - 11.33 \times age + 9.13 \times height + 26.88 \times weight - 0.12 \times weight^2$
VO_2 percentage predicted	$100 \times \frac{VO_{2\ (peak)}}{predicted\ VO_2}$

Supplemental Figure S*1*: Optimal number of clusters based on the selected cluster validity index (CVI). Left panel illustrates the score for each cluster for men and the right panel for women. Red dot in both panels represents the selected value and the respective score

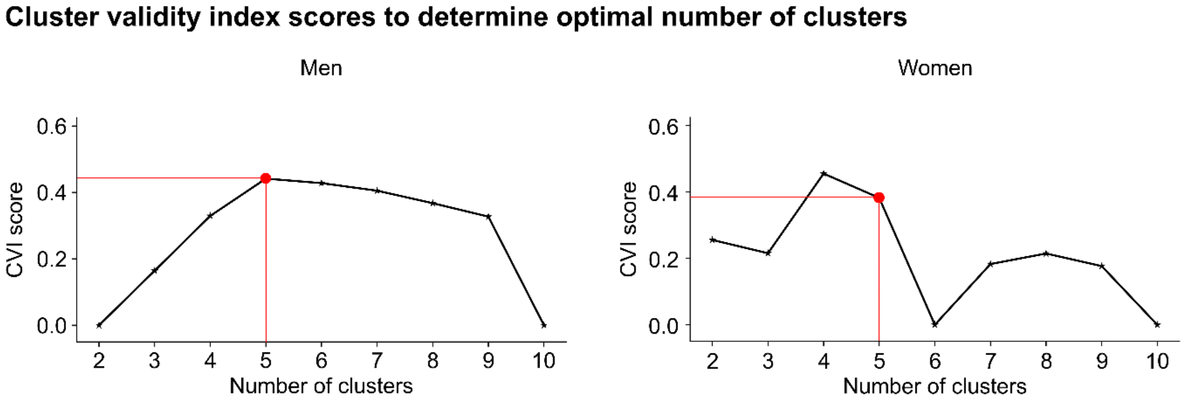

Supplemental Figure S*2*: Overview of the computational pipeline. Blue and orange parallelograms illustrate the input data and the results of the processing steps respectively. Green rectangles indicate the processing steps, while the flow of the steps are represented by the black arrows

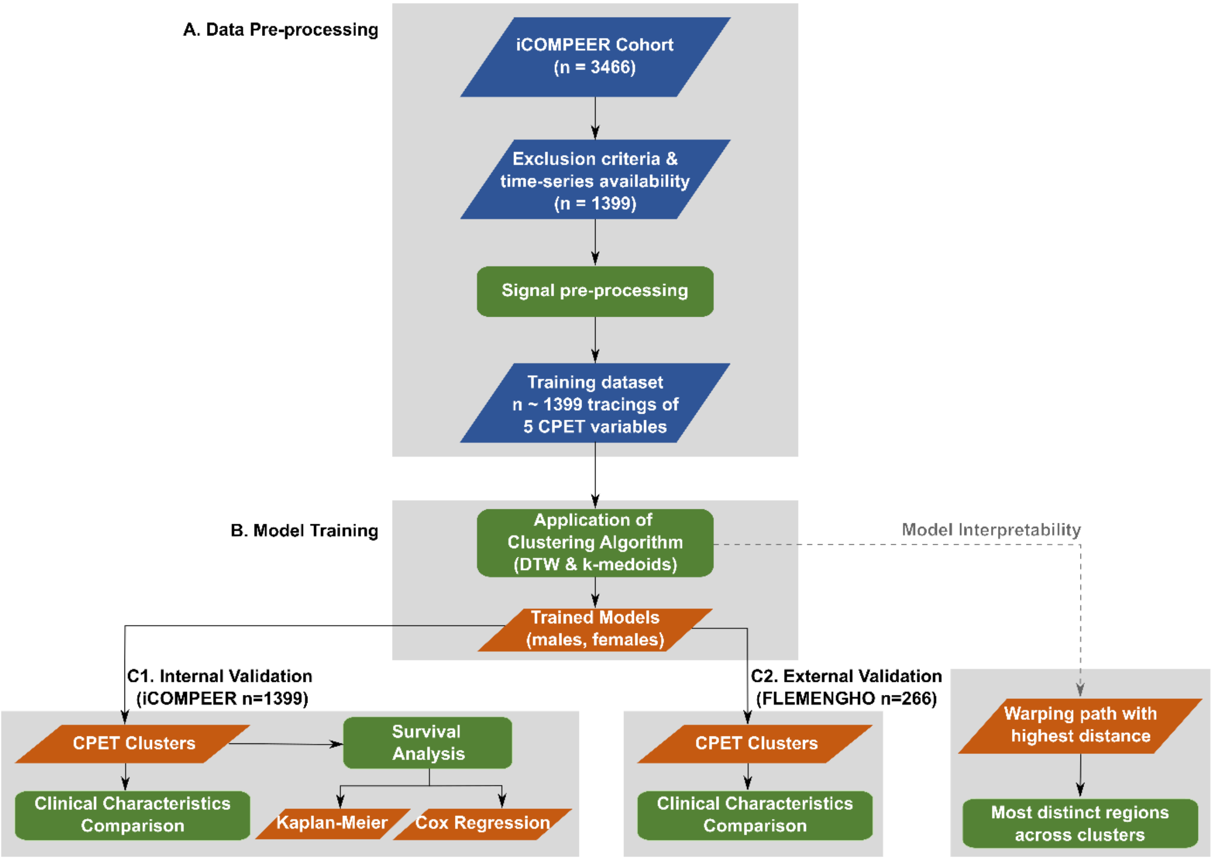

Supplemental Figure S*3*: Cross-correlation of time series CPET recordings with respect to all potential lags (temporal shifts). Each row and column corresponds to a specific CPET variable with the main diagonal showing the autocorrelation of the investigated parameters. In each sub-figure the x-axes show the time lag of the time series. We observed a higher correlation between V̇O2, V̇CO2, V̇E at small time-lags (or at zero time lag) and between RER and V̇CO2 for zero or small time lags.

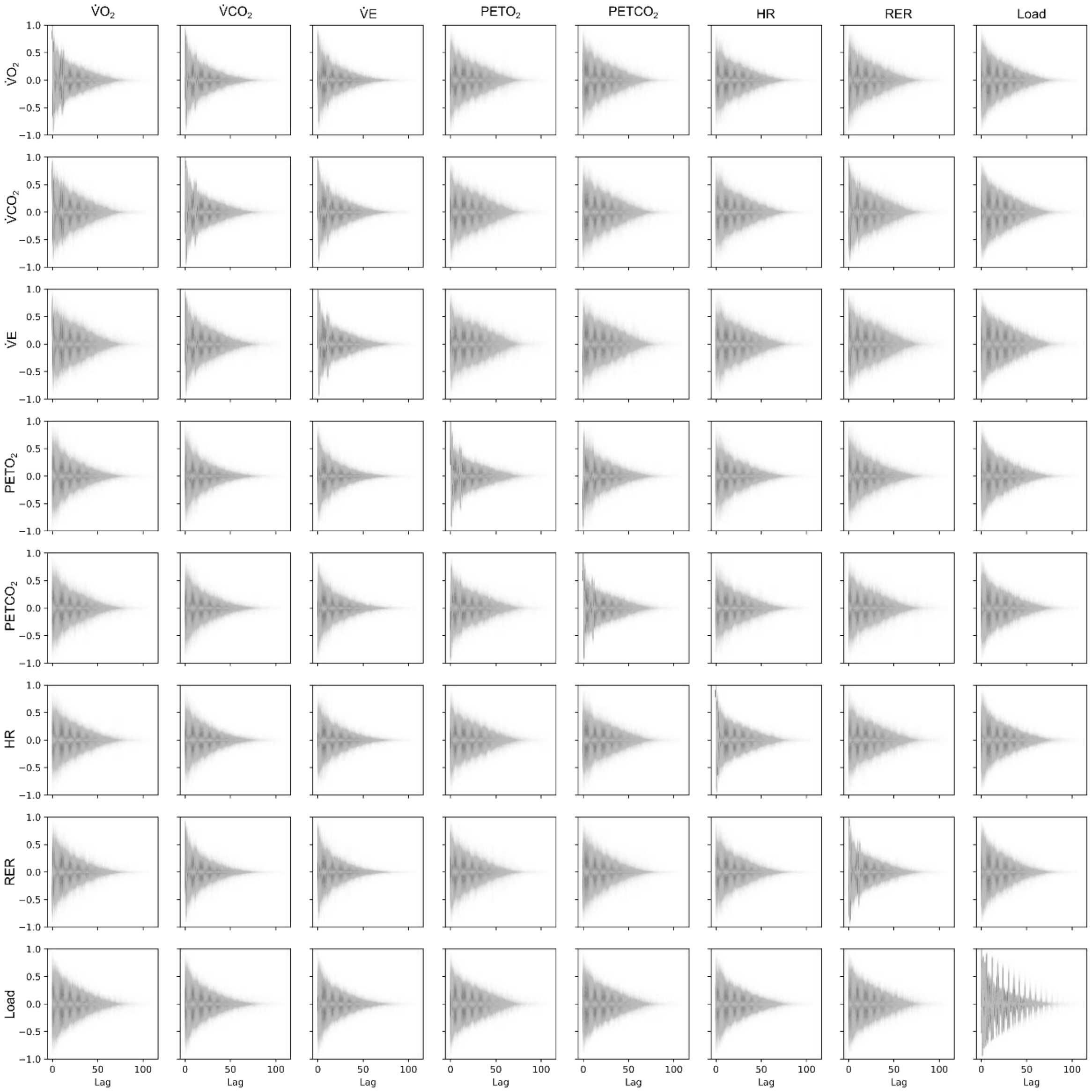

Supplemental Figure S4: Spearman's cross-correlation table for zero temporal lag. V̇O2 shows strong correlation with V̇CO2 and V̇E.

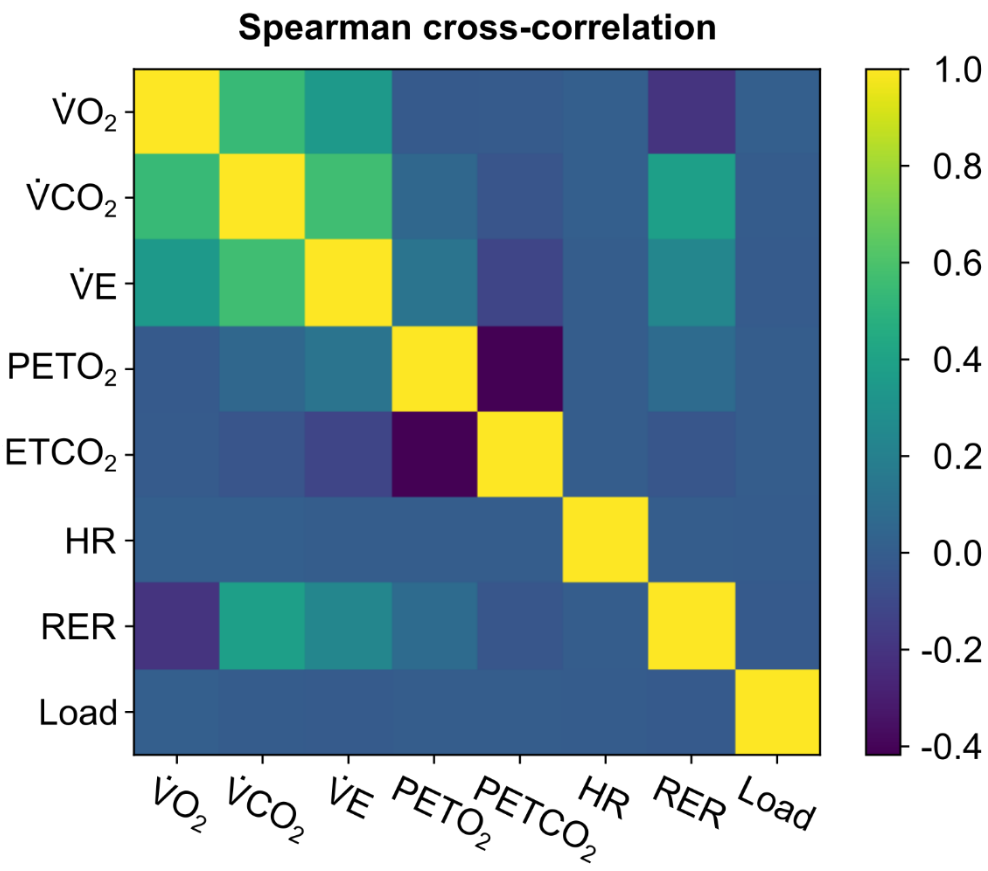

Supplement: Document S1. Figures S1–S4 and Tables S1–S5 [file mmc1.pdf]
